# 10-year survival of total ankle arthroplasties: A report on 780 cases from the Swedish Ankle Register

**DOI:** 10.3109/17453674.2011.636678

**Published:** 2011-11-25

**Authors:** Anders Henricson, Jan-Åke Nilsson, Åke Carlsson

**Affiliations:** ^1^Department of Orthopedics, Falun Central Hospital and Center for Clinical Research Dalarna, Falun; ^2^Department of Clinical Sciences and Orthopedics, Lund University, Department of Orthopedics, Skåne University Hospital, Malmö, Sweden

## Abstract

**Background and purpose:**

There is an ongoing need to review large series of total ankle replacements (TARs) for monitoring of changes in practice and their outcome. 4 national registries, including the Swedish Ankle Register, have previously reported their 5-year results. We now present an extended series with a longer follow-up, and with a 10-year survival analysis.

**Patients and methods:**

Records of uncemented 3-component TARs were retrospectively reviewed, determining risk factors such as age, sex, and diagnosis. Prosthetic survival rates were calculated with exchange or removal of components as endpoint—excluding incidental exchange of the polyethylene meniscus.

**Results:**

Of the 780 prostheses implanted since 1993, 168 (22%) had been revised by June 15, 2010. The overall survival rate fell from 0.81 (95% CI: 0.79–0.83) at 5 years to 0.69 (95% CI: 0.67–0.71) at 10 years. The survival rate was higher, although not statistically significantly so, during the latter part of the period investigated. Excluding the STAR prosthesis, the survival rate for all the remaining designs was 0.78 at 10 years. Women below the age of 60 with osteoarthritis were at a higher risk of revision, but age did not influence the outcome in men or women with rheumatoid arthritis. Revisions due to technical mistakes at the index surgery and instability were undertaken earlier than revisions for other reasons.

**Interpretation:**

The results have slowly improved during the 18-year period investigated. However, we do not believe that the survival rates of ankle replacements in the near future will approach those of hip and knee replacements—even though improved instrumentation and design of the prostheses, together with better patient selection, will presumably give better results.

The current generation of total ankle replacements, uncemented and avoiding rotational strain in a 3-component design with a polyethylene meniscus, have shown promising but varying results in medium-term and long-term reports (Kofoed 2004, Carlsson 2006, Doets and al. 2006, Wood et al. 2008, 2010, Bonnin et al. 2010, Karantana et al. 2010). National registries have a large number of patients operated on by different surgeons, and may thus give better insight into the current situation than what can be reported from a single surgeon or institution. 5-year prosthetic survival rates of between 0.78 and 0.88 have been reported by the Norwegian, Swedish, New Zealand, and Finnish national registries. (Fevang et al. 2007, Henricson et al. 2007, Hosman et al. 2007, Skyttä et al. 2010). Here we present longer-term follow-up of total arthroplasties reported to the Swedish Ankle Register.

## Patients and methods

The first of the third-generation total ankle replacements in Sweden were performed in 1993. Since then, all total ankle replacements are reported to a national register by each surgeon using a paper form. Hospital data, demographic data, date of index operation, date of revision surgery, operated side, diagnosis (primary or reason for revision), type of prosthesis, and—in cases of revision—the type of procedure are reported.

Between April 14, 1993, and June 15, 2010, 780 primary total ankle replacements were reported to the register (Table 1). Because of small numbers of the BP, AES, Mobility, and CCI prostheses—and due the similarity in design—these implants were analyzed together as a group called BP-type. The diagnoses were rheumatoid arthritis (RA) in 282 cases (36%), primary or idiopathic osteoarthritis (OA) in 185 (24%), posttraumatic arthritis (PtA) in 266 (34%), and various diagnoses including hemophilia, hemochromatosis, and psoriatic arthritis in 47 cases (6%) (Table 2). 31 patients with RA and 23 patients with other diagnoses had both ankles replaced.

**Table 1.** Numbers of ankle prostheses implanted in Sweden annually

Year	STAR	BP	AES	Hintegra	Mobility	CCI	Total
1993	6						6
1994	13						13
1995	11						11
1996	8						8
1997	24						24
1998	34						34
1999	25						25
2000	45	1					46
2001	48						48
2002	40	21	3	10			74
2003	25	23	17	14			79
2004	17	29	16	4			66
2005	18	13	23	2	9		65
2006	7	11	21	6	21		66
2007	1	7	18		20		46
2008		4	17		32	16	69
2009					31	40	71
2010 **^a^**					19	10	29
Total	322	109	115	36	136	66	780

**^a^** up to June 15

**Table 2.** Demographic data

	n	% females	Mean age (range)
RA	282	78	56 (18–86)
OA	185	48	62 (30–84)
PtA **^a^**	266	54	58 (25–86)
Other	47	47	56 (28–74)
All diagnoses	780	61	58 (18–86)

**^a^** posttrauma arthritis.

### Statistics

Survival curves were constructed according to Kaplan-Meier. Cox proportional regression was used to calculate hazard ratios (HRs) with 95% confidence intervals (CIs). The assumptions for Cox regression analysis were evaluated graphically in survival curves and log-minus-log plots and were estimated to be fullfilled. As endpoint, we used revision leading to exchange or extraction of 1 or more of the 3 prosthetic components with the exception of incidental exchange of the polyethylene insert (Henricson et al. 2011). 3 patients were revised bilaterally, and both ankles were included in the analyses. Inclusion of bilateral observations in arthroplasty studies appears to have a negligible effect on survival estimates (Ranstam and Robertsson 2010).

## Results

17 hospitals had reported to the Swedish Ankle Register, but at the time of writing total ankle replacement is only performed at 6 hospitals in Sweden.

168 ankles (22%) were revised (Table 3). 67 (40%) of them were revised because of loosening of the tibial and/or the talar component, and another 21 because of instability with or without dislocation of the polyethylene (PE) meniscus. 19 ankles were considered to be technical failures by the surgeons performing the revisions, with malpositioning of the tibial component—too lateral or too medial, or at an incorrect angle. Use of a tibial component that was too short from front to back was also considered to be a technical error. Severe pain for no obvious reason in 11 ankles eventually resulted in revision. In addition, 17 ankles were revised because of severe wear or fracture of the mobile bearing component.

**Table 3.** Reasons for revision

	Single-coated	Double-coated						
	STAR	STAR	BP	AES	Hintegra	Mobility	CCI	Total
Numbers	117	205	109	115	36	132	66	780
Aseptic loosening	33	16	4	5	3	2	4	67
Technical error	8	8		1	2			19
Instability	1	2	5	8	1	3	1	21
Infection	3	10	1	2	1	2		19
Intractable pain	4	6				1		11
PE breakdown/fracture	7	4	3	1		2		17
Painful varus		2	1	4	1		1	9
Fracture		1	3	1				5
Total	56	49	17	22	8	10	6	168

30 cases (4%) were complicated by septic arthritis. 19 of those required revision and were included when we calculated prosthetic survival. The remaining cases were treated with antibiotics, synovectomy, and washout.

The revison rate was 21–22% both in the unilateral and the bilateral ankles. There were 118 other secondary procedures that were not classified as revisions e.g. subtalar, triple, or talo-navicular fusions, debridement of the medial gutter, deltoid ligament release, and Achilles tendon lengthening.

**Figure 1.**
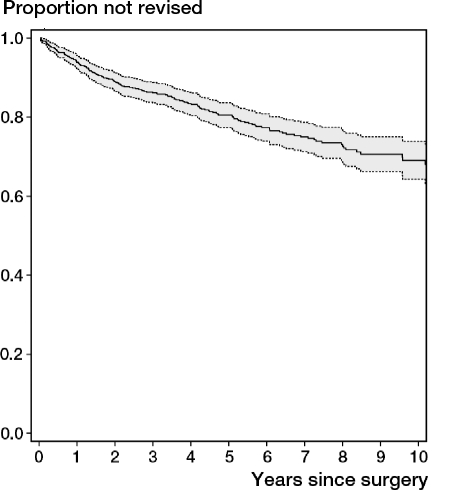
Estimated cumulative survival and 95% CI for all 780 arthroplasties.

**Figure 2.**
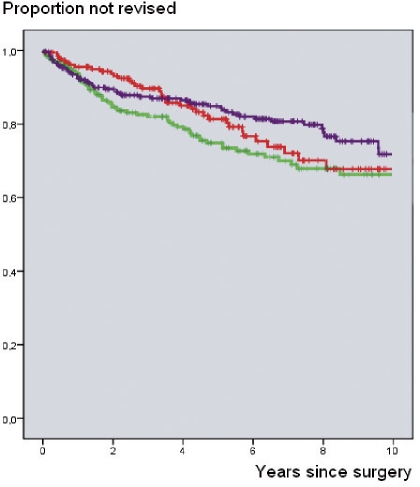
Estimated cumulative survival for ankles replaced due to rheumatoid arthritis (purple), osteoarthritis (red), and posttraumatic osteoarthritis (green).

**Figure 3.**
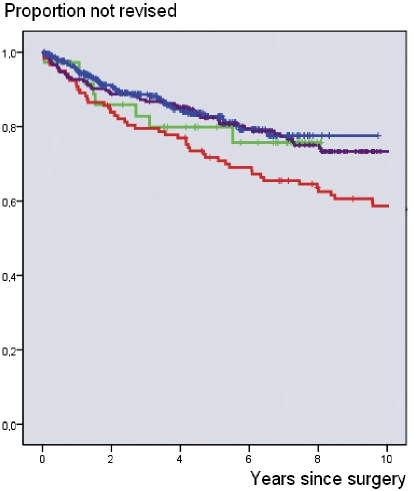
Estimated survival for the different designs implanted. The blue curve represents the BP-type designs, the green curve the Hintegra, the purple curve the double-coated STAR, and the red curve the single-coated STAR prostheses.

The estimated overall 5-year survival rate was 0.81 (CI: 0.79–0.83) and the 10-year survival rate was 0.69 (CI: 0.67–0.71) (Figure 1 and Table 4). For RA patients, the estimated survival rate at 10 years was 0.72, for OA patients it was 0.68, and for patients with posttraumatic arthritis it was 0.66 (Figure 2). Compared to OA patients, HR for RA patients was 0.9 (CI: 0.6–1.3) and compared to patients with posttraumatic arthritis it was 0.7 (CI: 0.5–1.0). No statistically significant difference was found between the BP-type prostheses (BP, AES, Mobility, and CCI (p = 0.24). The 10-year survival rate of the single-coated STAR was 0.58 (Figure 3). The prosthetic survival rate was statistically significantly lower for the single-coated STAR prostheses implanted between 1993 and 1999 than for the BP-type prostheses (HR = 1.7; CI: 1.1–9.5). The survival rates of the Hintegra and double-coated STAR prostheses were similar to that of the BP-type prostheses (Table 5).

**Table 4.** Life table

Interval (years)	No. entering the interval	No. of events	Cumulative proportion surviving at the end of the interval	95% CI
0–1	780	46	0.94	0.93–0.95
1–2	674	33	0.89	0.88–0.90
2–3	568	17	0.86	0.85–0.87
3–4	496	15	0.83	0.82–0.84
4–5	434	14	0.81	0.79–0.83
5–6	355	13	0.77	0.75–0.79
6–7	288	8	0.75	0.73–0.77
7–8	219	5	0.73	0.71–0.75
8–9	165	5	0.70	0.68–0.72
9–10	114	2	0.69	0.67–0.71

**Table 5.** Hazard ratio (HR) of survival rates for different prostheses

Prosthesis	HR	95% CI
BP-type	1	Reference
Hintegra	1.2	0.57–2.5
STAR I (single-coated)	1.7	1.1–2.5
STAR II (double-coated)	1.1	0.75–1.6

Patients younger than 60 years of age with OA or posttraumatic arthritis had a statistically significantly higher risk of revision than patients who were older than 60 years (HR = 1.8; CI: 1.3–2.8). This risk was statistically significant for women (HR = 2.0; CI: 1.2–3.6) but not for men. Age had no influence on the revision rate in patients with RA (HR = 1.4; CI: 0.8–2.4). Revisions due to technical error, infection, and instability were undertaken earlier than those undertaken as a result of component loosening and meniscal wear (Table 6).

**Table 6.** Time (years) and 95% CI from index surgery to revision

Reason for revision	Mean	95% CI
Valgus malalignment	1.0	0.2–1.8
Infection	1.2	0.6–1.8
Technical error	1.3	0.6–2.0
Fracture	1.4	0.3–3.0
Instability	1.8	0.9–2.7
Loosening tibia	2.5	1.7–3.3
Intractable pain	2.5	1.6–3.4
Varus malalignment	2.9	1.3–4.4
Loosening talus and tibia	5.3	4.0–6.5
PE breakdown/fracture	5.6	3.8–7.5
Loosening talus	7.8	6.3–9.2

## Discussion

The annual number of ankle replacements performed in Sweden (with 9 million inhabitants) increased until 2002, and then a steady state of around 70 per year was reached (Table 1). This indicates 1 replacement per 100,000 inhabitants over the age of 15. The corresponding figure is the same in Norway (http://nrlweb.ihelse.net) and Scotland (www.arthro.scot.nhs.uk) but twice as many replacements are performed annually in Finland (Skyttä et al. 2010) and 3 times as many are performed in New Zealand (www.cdhb.govt.nz). In Denmark, the corresponding figure is 2.8 (www.sst.dk) and in England and Wales it is 1.2 (www.njrcentre.org.uk).

Earlier reports from 4 national registries have shown a 5-year survival rate of 78–88% (Henricson et al. 2007, Fevang et al. 2007, Hosman et al. 2007, Skyttä et al. 2010). However, the definitions of a revision of a total ankle replacement were not identical in these reports. Here, for the first time, we present data from a national register for up to 18 years of follow-up of third-generation total ankle replacements. In our first report (Henricson et al. 2007) we found a survival rate of 78% (95% CI: 74–82) at 5 years for the total material. The endpoint for the calculations was exchange or removal of any of the components (Henricson et al. 2011).

In the present study, the overall 10-year survival (with identical endpoint) was estimated to be 69% (CI: 67–71). When we excluded the single- and double-coated STAR prostheses (for reasons given below), the 10-year survival was estimated to be 0.78 (CI: 0.72–0.83).

As a rule, specialized units report higher survival rates. Thus, Wood et al. (2008) found a 10-year survival rate of 0.80 (CI: 0.71–0.90) for 200 STAR prostheses. In smaller material, Doets et al. (2006) found a 10-year survival rate of 0.89 (CI: 0.82–97) for 74 BP prostheses and Karantana et al. (2010) found an 8-year survival rate of 0.84 (CI: 0.67–0.92) for 52 STAR prostheses. Bonnin et al. (2010) reported a 10-year survival rate of 0.85 (CI: 0.75–0.95) for 98 Salto prostheses.

In Sweden, units that were not satisfied with the results after having performed a limited number of replacements decided to perform arthrodeses only and referred candidates for replacement to specialized units. This may have contributed to better results in later years.

We found a statistically significant difference in performance of the single-coated STAR prosthesis compared with the other designs. It performed worse. The only published randomized study comparing different designs of total ankle replacements found better performance of the STAR prosthesis than of the BP prosthesis, but the difference was not statistically significant. That study included both single-coated and double-coated STAR prostheses (Wood et al. 2009). Also, 2 systematic reviews of the outcome of total ankle replacements have been published. Stengel et al. (2005) found the weighted 5-year survival of 1,107 third-generation total ankle replacements to be 0.91 (CI: 0.84–0.97). No significant differences between the prostheses included (STAR, ESKA, Ramses, LCS, and BP) were found. Gougoulias et al. (2010) reported an overall revision rate of 10% for 801 third-generation total ankle replacements (STAR, BP, HINTEGRA, Salto, and Mobility) and no superiority of any of the prostheses after a follow-up of 5 years.

Aseptic loosening of components was the most common reason for revision, and it was mainly a problem with the STAR prosthesis (Table 3). The STAR prosthesis was the first third-generation design to be introduced in Sweden, and most surgeons were therefore in a learning phase. Also, the STAR prosthesis is technically demanding and the instrumentation was inferior, at least during the first years. The STAR prosthesis is no longer used in Sweden (Table 1). The single-coated STAR is not manufactured any more.

As expected, technical mistakes at surgery, infections, and instability problems resulted in early revision. Meniscal wear and aseptic loosening, notably of the talar component, resulted in later revision. The reason may be that loosening of the tibial component can be seen more easily in radiographs than loosening of the talar component.

In the present study, the risk of revision was increased for patients younger than 60 years with primary osteoarthritis or posttraumatic osteoarthritis. This difference was only statistically significant for women. The revision rate in patients with rheumatoid arthritis was not influenced by age. The revision rate in younger patients was also elevated in our earlier report from the Swedish Ankle Register (Henricson et al. 2007), but not in the reports from the Norwegian, New Zealand, or Finnish registers (Fevang et al. 2007, Hosman et al. 2007, Skyttä et al. 2010). Younger patients have higher demands and a higher activity level than older patients, which might affect the longevity of a total ankle replacement.

The infection rate of 4% that we found involved all deep infections, including late hematogenous infections; the 2% that led to revision was at the same level as in other reports. Thus, Doets et al. (2006) found a rate of 3%, Raikin et al. (2010) found a rate of 3%, and Whalen et al. (2010) reported 7%. The anterior incision used for total ankle replacement surgery is considered to indicate a high risk of healing complications. Other risk factors are smoking, peripheral vascular disease, and cardiovascular disease (Whalen et al. 2010) but Raikin et al. (2010) only found inflammatory arthritis to be a statistically significant risk factor for major wound complications.

The surgical challenge in performing a total ankle replacement and the long learning curve is well known (Andersson et al. 2003, Carlsson 2006, Henricson and Ågren 2007), and in our earlier study we found that experience reduced the revision rate (Henricson et al. 2007). This study supports that finding, since the results have improved with time.

Bai et al. (2010) found that complications and secondary surgical procedures were higher in posttraumatic patients than in patients with primary osteoarthritis, but we found similar survival rates concerning different diagnoses (Figure 2).

An obvious limitation of reports from national registries is the uncertainty of adequate reporting from different centers. We are personally acquainted with every surgeon who performs total ankle replacement in Sweden. Furthermore, we have compared data with information from hospital records, and in recent years also with data from the Nation Health Authority. The reporting rate can therefore be considered to be complete.

Another limitation of our study is the problem of bilateral cases, which were included in our survival analyses. 3 patients were revised bilaterally. However, Ranstam and Robertsson (2010) reviewed the literature on the effect of including bilateral observations and found that all reports suggest a negligible effect on survival estimates.

It seems appropriate to inform presumptive patients that the probability of retaining an ankle arthroplasty of modern design for 10 years is about 80%. They should also be informed that when a total ankle replacement fails, there is the possibility of performing a successful ankle arthrodesis by various methods: intramedullary nailing through a femoral head autograft (Thomason and Eyres 2008), anterior plating (Plaass et al. 2009, Berkowitz et al. 2011), or the use of an intramedullary nail through a trabecular metal implant (Henricson and Rydholm 2010). Caution can no doubt be recommended with posttraumatic cases and with younger patients, especially younger women with osteoarthritis.
